# Validation of the theoretical domains framework for use in behaviour change and implementation research

**DOI:** 10.1186/1748-5908-7-37

**Published:** 2012-04-24

**Authors:** James Cane, Denise O’Connor, Susan Michie

**Affiliations:** 1 School of Psychology, Keynes College, University of Kent, Canterbury, Kent, CT2 7NP, UK; 2 School of Public Health and Preventive Medicine, Faculty of Medicine, Nursing and Health Sciences, Monash University, The Alfred Centre, 99 Commercial Road, Melbourne, VIC, 3004, Australia; 3 Research Department of Clinical, Centre for Outcomes Research and Effectiveness (CORE), Education and Health Psychology, University College London, 1-19 Torrington Place, London, WC1E 7HB, UK

**Keywords:** Theoretical domains framework, Behaviour, Change, Implementation, Validation, Theory

## Abstract

****Background**:**

An integrative theoretical framework, developed for cross-disciplinary implementation and other behaviour change research, has been applied across a wide range of clinical situations. This study tests the validity of this framework.

****Methods**:**

Validity was investigated by behavioural experts sorting 112 unique theoretical constructs using closed and open sort tasks. The extent of replication was tested by Discriminant Content Validation and Fuzzy Cluster Analysis.

****Results**:**

There was good support for a refinement of the framework comprising 14 domains of theoretical constructs (average silhouette value 0.29): ‘Knowledge’, ‘Skills’, ‘Social/Professional Role and Identity’, ‘Beliefs about Capabilities’, ‘Optimism’, ‘Beliefs about Consequences’, ‘Reinforcement’, ‘Intentions’, ‘Goals’, ‘Memory, Attention and Decision Processes’, ‘Environmental Context and Resources’, ‘Social Influences’, ‘Emotions’, and ‘Behavioural Regulation’.

**Conclusions:**

The refined Theoretical Domains Framework has a strengthened empirical base and provides a method for theoretically assessing implementation problems, as well as professional and other health-related behaviours as a basis for intervention development.

## **Background**

Behaviour change is key to improving healthcare and health outcomes. Behaviours may be those of healthcare workers, such as implementation of evidence-based practice, of patients, such as medication adherence, or of the general population, such as smoking cessation and increasing physical activity. Despite high-level recommendations to improve implementation of evidence-based practice [1,2] and a rapidly developing field of implementation science, implementation remains variable, with numerous organisational and individual factors influencing healthcare workers’ behaviour. These factors include the availability of evidence, its relevance to practice, the dissemination of evidence and guidelines, individual motivation, the ability to keep up with current changes, clarity of roles and practice, and the culture of specific healthcare practices [3,4].

Improving implementation of evidence-based practice by healthcare workers depends on changing multiple behaviours of multiple types of people (*e.g.*, health professionals, managers, administrators) [5]. Changing behaviour is not easy, but is more effective if interventions are based on evidence-based principles of behaviour change [6]. These principles form part of many theories of behaviour change, but are seldom drawn on in designing and evaluating implementation interventions. There is some evidence that behaviour change interventions informed by theory are more effective than those that are not [7,8]. Designing interventions on the basis of practitioner or researcher intuition rather than theory precludes the possibility of understanding the behaviour change processes that underlie effective interventions and of applying this knowledge to inform the design of future interventions. This is also the case where theory is cited but poorly applied to intervention development [9].

In a review of 235 guideline development and implementation studies, only 22.5% were judged to have used theories of behaviour change, and 16.6% of studies using a single theory [10]. A further 4.3% used only selected constructs from theories; across the majority of studies there was no clear rationale for theory use. While use of a single theory may be appropriate and lends itself to theory testing, in many cases the selection has not been justified and the theory is not tested [9]. If theory selection is not informed by a comprehensive theoretical assessment of the implementation or other behavioural problem, there is a risk of missing relevant theoretical constructs or including irrelevant ones. A second problem in applying theory to intervention design stems from basing interventions on several theories with overlapping theoretical constructs [11,12]. This makes it difficult to identify the specific processes underlying successful behaviour change.

To overcome such problems, an integrative framework of theories of behaviour change was developed by 18 psychological theorists in collaboration with 16 health service researchers and 30 health psychologists [13]. The aim of the Theoretical Domains Framework (TDF) was to simplify and integrate a plethora of behaviour change theories and make theory more accessible to, and usable by, other disciplines. The group identified 33 theories and 128 key theoretical constructs related to behaviour change and synthesised them into a single framework to assess implementation and other behavioural problems and inform intervention design. They used a six stage consensus approach: identifying theories and theoretical constructs relevant to behaviour change, where a theoretical construct was defined as ‘a concept specially devised to be part of a theory’ [13]; simplifying these resulting constructs into overarching theoretical domains, where a theoretical domain was defined as ‘a group of related theoretical constructs’ [13]; evaluating the importance of the theoretical domains; conducting an interdisciplinary evaluation and synthesis of the domains and constructs; validating the domain list; and piloting interview questions relevant to the constructs and domains. This resulted in 12 theoretical domains and exemplar questions for each to use in interviews or focus groups to provide a comprehensive theoretical assessment of implementation problems.

This framework has been used by research teams across several healthcare systems to explain implementation problems and inform implementation interventions. For example, in Australia it has been used to identify the barriers and enablers to the implementation of evidence-based guidelines for acute low back pain [14,15] and develop theory-informed behaviour change interventions [16]. In the UK, examples include studies of the barriers and levers related to hand hygiene [17]; the assessment of theoretical domains relevant to blood transfusion practice across different contexts including neonatal and adult intensive care units [18,19]; and identifying difficulties in implementing guidelines relating to schizophrenia [20]. In Denmark, it has been used to understand behaviour in the implementation of tobacco use prevention and counselling guidelines amongst dental providers [21]. Most of this research has used interviews and focus groups that are resource intensive; a questionnaire measure is currently being developed by the authors. This will facilitate research investigating prediction of implementation and other types of behaviour change.

This article is one in a series of articles documenting the development and use of the TDF to advance the science of implementation research. To inform future use of the TDF, we conducted the current study to provide a more thorough test of the validity of the framework than was carried out in the original research. The overall objective of the study was to examine the content validity of the TDF. Specifically, we wanted to confirm the optimal domain structure (number of domains), domain content (component constructs in each domain), and domain labels (most appropriate names that best reflected the content of the validated domain structure). Card sorting methodology was used to conduct the validation of the TDF in this study. By building on the validation process undertaken by Michie *et al*. [13] the present study aimed to improve the empirical basis of this framework.

## **Method**

### **Design**

The study used a cross-sectional design.

### **Participants**

Eligible participants possessed a good understanding of behaviour change theory and were unaware of the original framework reported in Michie *et al*. [13]. Potentially eligible participants were identified by systematically searching five online electronic journal databases (Web of Science, PsychInfo, CINAHL Plus, Ingenta Connect, JStor) using terms ‘behaviour change’ AND ‘theory’ from 1990 to 2011, by sending email invitations through membership mailing lists for the European Health Psychology Society, the American Psychological Association Division of Health Psychology, the USA’s National Institute of Health’s Behaviour Change Consortium, the Midlands Health Psychology Network in the UK, and by searching through delegate lists from the 2008 to 2010 annual conferences of the UK Society for Behavioural Medicine and British Psychological Society’s Division of Health Psychology. The contact details of all individuals identified as authors on papers identified through the electronic database searches were located via publically available sources (*e.g.*, searches of university and other organisation websites).

Of 101 individuals who asked for full information about the study, 61 expressed an interest in taking part and were sent links to one of the online tasks; 37 of these (61%) completed their assigned task. The majority were from the UK (16), with the remaining participants being from the Netherlands (8), USA (2), Ireland (2), Australia (2), Italy (2), Portugal (1), South Africa (1), Greece (1), Germany (1), and Switzerland (1). The 27 women and 10 men had a mean age of 36.54 years (range 22 to 62).

The sample size for the tasks was based on estimates of between six and 36 participants shown as sufficient for sort and cluster analysis tasks [22-28]. For content-validation tasks, such as those proposed in the closed sort task, two to 24 participants have been shown to be sufficient [29-32], with more than five participants reducing the influence of rater outliers [33].

### **Evaluating the framework**

To evaluate the original framework, a three step method was used:

Step one: Identify the optimal number of domains by sort task methods.

Step two: Establish domain content by identifying the most suitable construct allocation to each of the domains.

Step three: Finalise domain labels by identifying the most appropriate labels for new domains (labels for domains that replicated the original ones were retained).

### **Sort task methodology**

**Figure 1:**
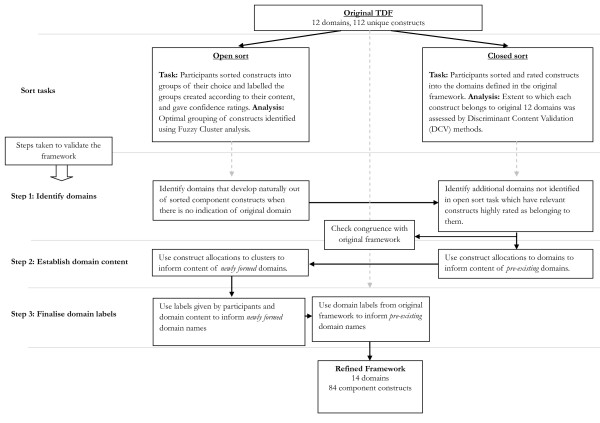
Steps taken to validate the Theoretical Domains Framework.

Two types of sort task were used: an open sort task and a closed sort task (see Figure 1). In the open sort task, participants were asked to sort constructs into groups of their choice and label these groups according to their content. The optimal grouping of constructs into domains was identified using Fuzzy Cluster Analysis [34], whereby sorting patterns across individual participants could be aggregated into clusters. This cluster technique has the benefit over the more commonly used k-means and k-medoid cluster analysis, and other grouping methods, in that it allocates a membership value (in the form of a probability value) for each possible construct-cluster pairing rather than simply assigning a construct to a single cluster, thereby the membership of items to more than one group could be assessed. The results obtained from the open sort task and Fuzzy Cluster Analysis were used to identify the optimal domain structure (step one), the content of new domains (step two), and the most appropriate domain labels, based on the group names given by participants (step three). In the closed sort task, participants were asked to sort constructs into the domains defined in the original framework and rate their confidence in their allocation of each construct to a domain. The extent to which participants believed each construct belonged to the original 12 domains was assessed by Discriminant Content Validation (DCV) methods. DCV methods are able to examine the confidence of relationship between a single item and a particular domain [35]. The results from the closed sort task were used to identify any domains containing constructs with high confidence ratings and good agreement between participants (step one), and the constructs allocated to these domains (step two). Both types of sort task informed step one because it was considered important to include domains that developed naturally from the construct groupings (as informed by the open sort task), and to include domains to which there was good agreement across participants in the confidence of construct allocation to these domains (as informed by the closed sort task). To achieve this, the open sort task results were used to identify the domains based on the clusters formed in the open sort task; the closed sort results were then used to identify any additional domains for which there was good agreement and confidence in assigning constructs to these domains.

## **Materials**

There were 112 unique constructs (see Additional file 1), after 12 duplicates from the original framework were removed (participants had the opportunity to sort each construct to multiple domains). Definitions for the domains and constructs were selected or constructed from dictionaries, (*e.g.*, American Psychological Association Dictionary of Psychology [36]), and internet sources (*e.g.*http://www.oed.com). Each definition was evaluated by the authors of the original framework and definitions were agreed by consensus. The sort tasks were delivered via an online computer program with constructs displayed at the top of the computer screen. For the open sort task, 24 unlabelled boxes were displayed below the construct item window into which the participants could sort the constructs. Above each box a space was given so that labels and descriptions for each group created could be given. For the closed sort task, 12 labeled boxes were displayed, each described by a single domain label from the original framework. In both tasks, individual constructs could be assigned to multiple boxes and for every allocation a confidence rating was requested using a drop-down menu (from 1 – ‘not at all confident’ to 10 – ‘extremely confident’). Constructs were presented in random order that was determined by the online program for each participant. Definitions for each construct (open and closed sort tasks) and domain (for closed sort task only) were available when the participant hovered over the word with their mouse. Participants were asked, through open-ended questions, to record the length of time they had been involved in using behaviour change theories, the context in which they used them (*e.g.*, teaching, research, etc.) and their expertise in behaviour change theory and in using behaviour change interventions (1 – ‘A great deal’, 2 – ‘quite a lot’, 3 – ’some’, 4 – ‘a little’, 5 – ’none’).

### **Procedure**

Invitations were emailed to potentially eligible participants giving a brief overview of the study and inquiring as to their expertise. If they considered themselves to have expertise in behaviour change theory and reported not knowing about the original framework, they were invited to participate and emailed the relevant web link to the task they were allocated to. Eligible participants were alternately allocated to an open or closed sort task based on the order in which they contacted the researchers. To avoid contamination of results across tasks, each participant was allocated to, and completed, only the closed sort task or the open sort task. For both tasks, an information screen gave a brief background to the study and asked for consent to take part. Participants were given detailed instructions on how to complete their task (see Additional file 2) before completing the sort task they were assigned to. There was no time limit. In both tasks, participants were asked to familiarise themselves with the construct definitions and, in the closed sort task only, the domain definitions. In the open sort task, participants were asked to sort the constructs into groups based on their semantic similarity using as many groups as they wanted to (up to 24) and were asked to provide a label for each group created. Participants could also provide a description for each group if they felt it was necessary. In the closed sort task, participants were asked to assign each construct to one or more of the 12 labelled domain boxes that they thought were most appropriate. Across both tasks, participants were asked to give confidence ratings for each assignment; if an item was not allocated to a domain it automatically received a confidence rating of 0. For both tasks, participants were made aware that they could allocate each construct to multiple groups. After assigning all constructs, participants were asked to review their construct allocations and to change any allocations if they wished to. On completion, participants were given further information about the project.

### **Data analysis**

Data were collected using MySQL databases. For the open sort task, data were the construct-group allocations, confidence ratings, and group labels allocated by the participant. For the closed sort task data were the construct-group allocations and confidence ratings.

### **Open sort**

To examine the optimal clustering of constructs (step one: identify domains), the open sort data were first organised into a dissimilarity matrix for each participant. Construct pairs, consisting of all possible construct-by-construct combinations, were assigned 0 if they were placed in the same group and 1 if they were placed in a different group. Agreement across these individual matrices was assessed using Mantel Correlations and Kendall's Coefficient of Concordance, *W*[37] using CADM.global and CADM.post from the ‘ape’ package [38] in the R statistics program [39]. Mantel Correlations determine the extent to which an individual participants’ matrix correlates with other participants’ matrices and were used to identify any potential outlying sort patterns that should be excluded from subsequent analysis. An individual’s matrix is considered to be an outlier when it negatively correlates with the other participants’ matrices [40]. Kendall’s Coefficient of Concordance provides an indication of the overall concordance across all participants’ sort patterns, Kendall’s *W* ranges from 1 to 0 [37], where 1 equals complete agreement in sorting patterns and 0 equals no agreement across sorting patterns. To identify the clusters formed through these sorting patterns, means were calculated for each construct pairing across individual matrices to form a single, aggregated dissimilarity matrix. Fuzzy Cluster Analysis of this matrix, using the FANNY algorithm [34,41] in the R statistics program, led to a membership value assigned to each construct-cluster pairing. These membership values, converted into percentages, serve as an indication of the extent to which a construct belongs to a particular cluster. Values near 100% indicate a high probability of association with a cluster and values near 0% indicate a low probability of association. Using these values, construct membership to multiple domains can be assessed (*e.g.*, construct x might have 53% membership to cluster y and 47% membership to cluster z).

Constructs were then allocated to the cluster with which it has the highest membership value (known as a ‘hard’ cluster solution and comparable to outputs of the k-means and k-medoid cluster methods). The fit of constructs within the clusters was calculated by silhouette values (*s(i)*) [42]. Silhouette values are calculated for each construct and range from +1, indicating strong association with a cluster and distance from neighboring clusters, through 0, indicating no distinct association with clusters, to −1, indicating that a construct is probably assigned to the wrong cluster and should be considered as belonging to the neighbouring cluster [42]. The average silhouette values (ave *s(i)*) across construct items within a cluster indicates how well a cluster is defined, and the overall average of silhouette values across clusters can be used to compare cluster solutions of different sizes.

The optimal outcome of the cluster analysis is to achieve the highest average silhouette value with the fewest clusters. It has been argued that average cluster silhouette values greater than 0.70 indicate a strong structure, whilst average silhouette values below 0.50 indicate weak structures and silhouette values <0.25 indicate that there is little evidence for any reliable structure [34]. Informed by these cutoff values, we considered that a construct with a silhouette value <0.25 in relation to a cluster did not belong to that cluster.

In addition to identifying the optimal domain structure, the open sort results were used to identify the extent to which the clusters replicated the construct allocation in the original framework when domain labels were not provided (step two: establish domain content). Congruence was quantified as the percentage of constructs from the original framework domain remaining in a cluster solution (*e.g.*, if domain *m* contained constructs *x*, *y*, and *z* and the cluster contained only *x* and *z*, then congruence was 67%). If the structure of the domains identified in the Fuzzy Cluster Analysis was considerably different from that of the original framework, confidence ratings would be used for secondary analysis to infer construct allocation to the new domains formed.

The group labels given by participants in the open sort task were organised according to their similarity and the frequency that they occurred across participants noted. Those labels that occurred frequently and were related to the content of the newly-formed domains were used to inform newly-formed domain labels (step three: finalise domain labels).

### **Closed sort**

To identify pre-existing domains that might also be considered for inclusion in the framework (step one: identify domains), the strength and agreement of construct allocations to pre-existing domains from the closed sort task were examined. Confidence ratings for each construct x domain pairing, excluding those that had no confidence ratings, were applied to a table. To examine the agreement of these construct x domain ratings and construct assignment across participants, two-way intraclass correlation coefficient (ICC) measures of consistency were used within each domain [43]. In line with previous research we classified ICC values <0.21 as indicating poor agreement, values between 0.21 to 0.40 as fair agreement, values between 0.41 to 0.60 as moderate agreement, and values of ≥0.61 as good to excellent agreement [44]. ICC values were used as an indication of the agreement in assignments and ratings across participants, but were not used to influence the final domain content.

To identify the strength of construct assignment to particular domains, DCV methods were used with one-sample t-tests on the participants’ confidence ratings against the value zero. A construct was considered as belonging to a domain if its mean confidence rating across participants was significantly greater than zero (*p* < 0.05) following the adoption of Hochberg’s correction [45] (see [29,35] for similar methods). Hochberg’s correction was used to control for the family-wise error rate given the number of tests used. Whilst this approach may not be considered a conventional use of one-sample t-tests, it provides a suitable criterion for inclusion and exclusion of constructs to a particular domain over and above the use of a subjective cut-off value. To ensure that domains with highly-rated, relevant constructs assigned to them were considered for inclusion in the framework, domains containing two or more constructs with ratings significantly greater than zero were considered. These constructs were also used to inform construct allocation to pre-existing domains (step two: establish domain content). The allocation of constructs to domains in the closed sort task was compared with construct allocation in the original framework to identify the extent of congruence between assigned constructs when domain labels were available. Here congruence was quantified as the percentage of constructs from the original framework domain that were also in that domain within this study.

### **Ethical approval**

The study was approved by University College London’s Psychology Department Ethics Committee [STF/2007/003], and each participant gave full informed consent prior to participating.

## **Results**

Eighteen participants completed the closed sort task and 19 the open sort task. All participants indicated that they had experience of behaviour change theory through either research, clinical practice, or teaching (or a combination of these). Participants reported working with behaviour change theory for a mean of 9.74 (SD = 9.14) years and rated both their expertise in behaviour change theory and in delivering behaviour change interventions as 1.97 (SD = 0.64) and 2.46 (SD = 0.90), respectively, as measured on five-point scales (lower score indicates more expertise).

### **Sample size suitability and open sort pattern concordance**

Post-hoc power analysis for the closed sort task revealed that there was sufficient power (82%) with the final sample size of 18 to detect a mean rating of 1.53 (SD = 2.42, d = 0.63) as significant within a one-tailed one-sample t-test with α = 0.05. The mean rating used in the power analysis was based on the mean of confidence ratings across all variables included in the closed sort analyses.

For the open sort task, Mantel Correlation analysis indicated that all participants’ matrices were positively correlated, with aggregated Mantel correlation values for each participant ranging from 0.14 to 0.25 (see Additional file 3). Therefore none of the participants’ sort patterns were considered as outliers, and matrices from all 19 participants were included in the final analysis. The overall concordance of sorting patterns was *W* = 0.22, *p* = 0.01, reflecting the unconstrained nature of this task and its high number of variables.

#### ***Step one: identify domains***

**Figure 2:**
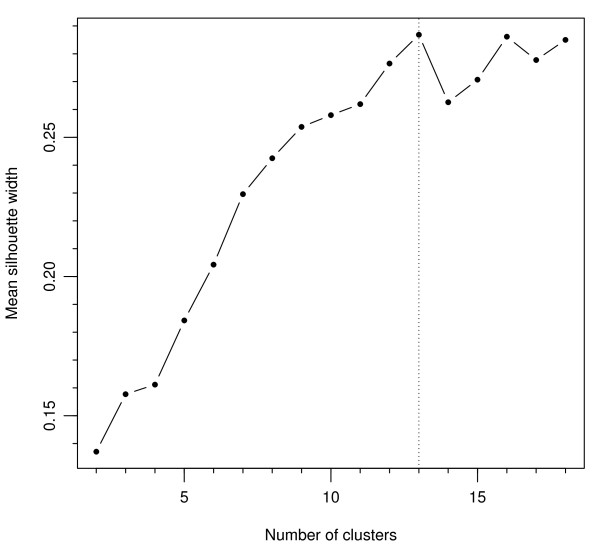
Comparison of fit across 2–18 cluster solutions.

In the open sort task, participants created on average 13.59 (SD = 3.61) groups. To identify the optimal fit for the cluster patterns based on the groups created by the participants, silhouette values for solutions of minimum two and maximum 18 clusters were examined. Analysis revealed the 13-cluster solution to be the most appropriate fit, achieving the highest overall average silhouette value of 0.29 (Figure 2 shows the relative overall silhouette values plotted for each cluster solution). The construct allocation within the ‘hard’ version of the 13-cluster solution, whereby each construct is allocated to only one domain, is presented in Table 1 next to the domains they most closely represent (see ‘Open sort task construct clusters’ and see Additional file 4 for related silhouette values).

**Table 1:** Comparison of the refined framework, closed sort task, and open sort task groupings

Refined framework domain name and constructs (* = new domain)	**Closed Sort Task construct groups (constructs achieving p < .05**^**a**^**; in order of confidence rating high – low)**	**Open Sort Task construct clusters (constructs in order of s(i) values decreasing;*italics*** **= constructs with silhouette value < .25)**
**1. Knowledge**		
Knowledge (including knowledge of condition /scientific rationale)	Knowledge (including knowledge of condition /scientific rationale)	-No cluster representing Knowledge-
Procedural knowledge	Procedural knowledge	
Knowledge of task environment	Knowledge of task environment	
**2. Skills**		
Skills	Skills	Competence
Skills development	Skills development	Skills
Competence	Competence	Skill assessment
Ability	Ability	Ability
Interpersonal skills	Interpersonal skills	Interpersonal skills
Practice	Practice	Skills development
Skill assessment	Skill assessment	Procedural knowledge
**3. Social/ Professional Role and Identity**		
Professional identity	Professional identity	Organisational development
Professional role	Professional role	Organisational culture / climate
Social identity	Social identity	Management commitment
Identity	Identity	Professional role
Professional boundaries	Professional boundaries	Crew resource management
Professional confidence	Professional confidence	Leadership
Group identity	Leadership	Change management
Leadership	Group identity	Professional boundaries
Organisational commitment	Organisational commitment	Organisational commitment
		Supervision
		Professional identity
		Project management
		*Champions / To champion*
		*Team working*
		*Power*
		*Hierarchy*
**4. Beliefs about Capabilities**		
Self-confidence	Self-confidence	Self-efficacy
Perceived competence	Perceived competence	Perceived competence
Self-efficacy	Self-efficacy	Self-confidence
Perceived behavioural control	Perceived behavioural control	Perceived behavioural control
Beliefs	Self-esteem	Professional confidence
Self-esteem	Beliefs	*Self-esteem*
Empowerment	Empowerment	
Professional confidence	Professional confidence	
5. Optimism*		
Optimism		Optimism
Pessimism		Pessimism
Unrealistic optimism		Unrealistic optimism
Identity		Identity
		*Mindsets*
**6. Beliefs about Consequences**		
Outcome expectancies	Outcome expectancies	Beliefs
Chars. of outcome expectancies ^b^	Chars. of outcome expectancies ^b^	Attitudes
Beliefs	Beliefs	Outcome expectancies
Anticipated regret	Anticipated regret	Chars. of outcome expectancies ^b^
Consequents	Consequents	*Illness representations*
7. Reinforcement *		
Rewards (proximal/distal, valued/not valued, probable/improbable)		Rewards (proximal/distal, valued/not valued, probable/improbable)
Incentives		Incentives
Punishment		Punishment
Consequents		Sanctions
Reinforcement		Contingencies
Contingencies		Reinforcement
Sanctions		Consequents
8. Intentions*		
Stability of intentions	Goals (autonomous, controlled)	Stability of intentions
Stages of change model	Intrinsic motivation	Stages of change model
Trans. model/stages of change ^b^	Goal target /setting	Trans. model/stages of change ^b^
	Distal and proximal goals	*Certainty of intentions*
	Goal priority	*Intention*
	Intention	*Commitment*
	Stability of intentions	*Intrinsic motivation*
	Certainty of intentions	*Mods. of the intention-behaviour gap*^b^
9. Goals*		
Goals (distal / proximal)		Goal target/ setting
Goal priority		Goals (distal / proximal)
Goal / target setting		Goal priority
Goals (autonomous / controlled)		Goals (autonomous / controlled)
Action planning		Action planning
Implementation intention		Implementation intention
		*Representation of tasks*
**10. Memory, Attention and Decision Processes**		
Memory	Memory	Memory
Attention	Attention	*Attention control*
Attention control	Attention control	*Attention*
Decision making	Decision making	*Decision making*
Cognitive overload / tiredness	Cognitive overload / tiredness	*Appraisal*
		*Schemas*
		*Cognitive overload / tiredness*
**11. Environmental Context and Resources**		
Environmental stressors	Environmental stressors	*Conflict-comp. demands, conf. roles*^b^
Resources / material resources	Resources / material resources	*Barriers and facilitators*
Barriers and facilitators	Barriers and facilitators	*Environmental stressors*
Organisational culture /climate	Organisational culture climate	*Knowledge of task environment*
Person x environment interaction	Person x environment interaction	*Person x environment interaction*
Salient events / critical incidents	Salient events / critical incidents	*Control of behaviour, material and social environment*
		*Knowledge*
		*Empowerment*
		*Negotiation*
		*Anticipated regret*
		*Threat*
		*Past behaviour*
**12. Social Influences**		
Social pressure	Social pressure	Group norms
Social norms	Social norms	Group conformity
Group conformity	Group conformity	Group identity
Social comparisons	Social comparisons	Social pressure
Group norms	Group norms	Social norms
Social support	Social support	Social support
Intergroup conflict	Intergroup conflict	Alienation
Power	Power	Social comparisons
Group identity	Group identity	Intergroup conflict
Alienation	Alienation	Social identity
Modelling	Modelling	
**13. Emotion**		
Anxiety	Anxiety	Anxiety
Fear	Fear	Depression
Affect	Affect	Positive / negative affect
Stress	Stress	Stress
Depression	Depression	Fear
Positive / negative affect	Positive / negative affect	Affect
Burn-out	Burn-out	Burn-out
**14. Behavioural Regulation**		
Self-monitoring	Self monitoring	*Learning*
Breaking habit	Breaking habit	*Review*
Action planning	Action planning	*Breaking habit*
		*Direct experience*
		*Self-monitoring*
		*Evaluation*

Key: ^a^ = after applying Hochberg’s correction for multiple comparisons within each domain, ^b^ - Chars. of outcome expect. = Characteristics of outcome expectancies; Conflict-comp. demands, conf. roles = Conflict - competing demands, conflicting roles; Mods. of the intention-behaviour gap = Moderators of the intention-behaviour gap; Trans. model/stages of change = Transtheoretical model and stages of change.

Within the 13 cluster solution, three of the original domains, ‘Beliefs about Capabilities’, ‘Beliefs about Consequences’, and ‘Motivation and Goals’, formed two clusters each. Four of the 13 clusters showed low average silhouette values (<0.25), one of the clusters arising from the ‘Motivation and Goals’ domain, also ‘Memory, Attention, and Decision Processes’, ‘Environmental Context and Resources’, and ‘Behavioural Regulation’. This was due to the inclusion of a number of constructs that had low (or negative) silhouette values, indicating that these constructs were not closely grouped with the other constructs within these clusters. To examine the impact of these low value constructs, they were removed and the average silhouette values of the clusters were recalculated. After removal, 10 clusters had average silhouette values greater than 0.25 (see Additional file 4, column 7) with the average silhouette value across these 10 domains equal to 0.47 and the concordance across sorting patterns increasing to *W* = 0.34 (*p* = 0.01). Three clusters remained with silhouettes below 0.25, ‘Environmental Context and Resources’, ‘Memory, Attention, and Decision Processes’, and ‘Behavioural Regulation’. Whilst these clusters showed relatively weak cluster formations in the open sort, the confidence ratings in the closed sort indicated that when the domain labels were apparent the confidence ratings of allocated constructs were sufficient to form domains. Therefore, these three domains were considered important to retain in the framework. Also, there was no cluster indicative of the domain of ‘Knowledge’ in the 13 cluster solution, with all constructs from the original ‘Knowledge’ domain allocated to alternative clusters; the constructs ‘Knowledge’ and ‘Knowledge of task environment’ were allocated to the ‘Environmental Context and Resources’ cluster, ‘Mindsets’ was allocated to one of the clusters arising from the ‘Beliefs about Capabilities’ domain, ‘Schemas’ was allocated to the ‘Memory, Attention and Decision Processes’ cluster and ‘Procedural knowledge’ was allocated to the ‘Skills’ cluster. However, within these construct reassignments only ‘Procedural Knowledge’ attained a silhouette value equal to or greater than 0.25 (all other knowledge-related constructs <0.23). In contrast, within the closed sort task the confidence ratings of three knowledge-related constructs, ‘Knowledge’, ‘Knowledge of Task Environment’ and ‘Procedural Knowledge’ indicated that knowledge might form a separate domain if the label ‘Knowledge’ was available (confidence ratings >6.32 across these three constructs). Therefore, it was considered that the ‘Knowledge’ should be included when it was thought to be important in the specific context.

Based on the results across both tasks, 14 domains were specified through this first step. Eight domains were similar to the original framework domains: ‘Knowledge’, ‘Skills’, ‘Social/Professional Role and Identity’, ‘Memory, Attention and Decision Processes’, ‘Environmental Context and Resources’, ‘Social Influences’, ‘Emotion’, and ‘Behavioural Regulation’. The domains ‘Beliefs about Capabilities’, ‘Beliefs about Consequences’, and ‘Motivation and Goals’ were retained but were divided into six new clusters. The domain of ‘Nature of the Behaviours’ was removed because it was not represented in the open sort by any single cluster solution and only had one construct assigned to it in the closed sort task.

#### ***Step two: establish domain content***

The mean confidence ratings and ICCs for the construct allocation to domains given in the closed sort task are shown in Additional file 4. In the closed sort task, the content of domains for ‘Emotion’, ‘Skills’, ‘Motivation and Goals’, ‘Social/Professional Role and Identity’, ‘Beliefs about Capabilities’, and ‘Memory, Attention and Decision Processes’ all showed good congruence with the constructs listed in the domains of the original framework (>69%) and fair ICCs (0.31 to 0.40). The domains of ‘Knowledge’, ‘Environmental Context and Resources’ and ‘Social Influences’ showed lower congruence with the constructs listed in the original domains (27% to 50%) and fair ICCs (0.26 to 0.39). The domains of ‘Behavioural Regulation’, ‘Nature of the Behaviours’, and ‘Beliefs about Consequences’ showed both low congruence between the original constructs and those assigned to these domains (<27%) and low ICCs (0.07 to 0.25). This was due in part to the low number of constructs assigned to these domains. ‘Behavioural Regulation’ only had two constructs out of the original ten (Self-monitoring, and Action planning) that were rated as belonging to the domain. ‘Nature of the Behaviours’ only had one construct (Routine/automatic/habit) included from the original six constructs. ‘Beliefs about Consequences’ only had five of the constructs included from the original framework. Only constructs that achieved significance in the closed sort after Hochberg correction were allocated to these pre-existing domains from the original framework.

For the newly formed clusters arising from ‘Motivation and Goals’ (two clusters), ‘Beliefs about Capabilities’ (one cluster), and ‘Beliefs about Consequences’ (one cluster), construct allocation was informed by the constructs assigned to these clusters in the open sort task that achieved individual construct silhouette values greater than 0.25.

To identify if any constructs should be considered for multiple allocation to domains the membership values from the Fuzzy Cluster Analysis were examined. This revealed that the majority of constructs (74/112) were strongly associated with only one cluster (*i.e.*, showed membership values over 80% to one specific cluster, see Additional file 5). A further 32 constructs showed moderately high associations with one cluster (memberships values between 28% to 79%), with the remaining proportion of memberships for these constructs spread over other clusters. Only eight constructs had the greatest proportion of their membership values split across at least two clusters, indicating possible multiple domain memberships, these were ‘Knowledge’, ‘Coping strategies’, ‘Empowerment’, ‘Anticipated regret’, ‘Negotiation’, ‘Moderators of the intention-behaviour gap’, ‘Routine/automatic/habit’, and ‘Past behaviour’. However, none of the multiple memberships indicated in the open sort results were replicated in the closed sort task where three different constructs, ‘Professional confidence’, ‘Beliefs’, and ‘Group identity’, were allocated to multiple domains. Given lack of agreement across the two tasks, only the multiple allocations shown in the closed sort task or multiple allocations that occurred through the construct selection process (*i.e.*, using the closed sort for predefined domains and using the open sort for new domains) were used in the final framework. Using this approach, six constructs were allocated to more than one domain: (the domains that constructs are allocated to are shown in parenthesis) ‘Action planning’ (Goals and Behavioural Regulation), ‘Beliefs’ (Beliefs about Consequences and Beliefs about Capabilities), ‘Consequents’ (Beliefs about Consequences and Reinforcement), ‘Group identity’ (Social/Professional Role and Identity and Social Influences), ‘Identity’ (Social/Professional Role and Identity and Optimism), and ‘Professional confidence’ (Social/Professional Role and Identity and Beliefs about Capabilities).

#### ***Step three: finalise domain labels***

Fifteen of the 19 open sort participants provided labels for the groups they created. The majority of labels were similar to those in the original framework: (number of participants giving that label shown in parenthesis): Knowledge (4), Skills (5), Intentions (7), Goals (6), Emotion (9), Cognitive-related (8), Beliefs (5), Beliefs about Capabilities (7), Outcomes (6), Environment-related (6), Organisational (7), Models / Theories (8), Learning / Reinforcement (7), Self-Regulation (3), Consequences (3), Social / Group (14), and Planning (2). Examples of other labels that could not be categorized (*i.e.*, labels given by only one participant) included ‘Techniques’, ‘Barriers’, ‘Awareness’, ‘Reviewing’, and ‘Persistence’. Given the similarity between the labels provided in the open sort task and the labels used in the original framework, those domains that were retained with only minor modification were allocated their respective label used in the original framework. The labels for the newly developed domains were based on the frequency of labels and the domain content: these were Intentions, Goals, Reinforcement, and Optimism. The domain label of ‘Emotion’ was pluralised to ‘Emotions’ to bring in line with the other domain labels and to ensure that it clearly represented the range of emotions that were included as component constructs. Therefore, the final labels chosen to represent the 14 domains were: ‘Knowledge’, ‘Skills’, ‘Social/Professional Role and Identity’, ‘Beliefs about Capabilities’, ‘Optimism’, ‘Beliefs about Consequences’, ‘Reinforcement’, ‘Intentions’, ‘Goals’, ‘Memory, Attention and Decision Processes’, ‘Environmental Context and Resources’, ‘Social Influences’, ‘Emotions’, and ‘Behavioural Regulation’.

### **The refined framework**

The refined framework contains 14 domains and 84 component constructs (the number of component constructs in each domain is defined in brackets): ‘Knowledge’ (3), ‘Skills’ (7), ‘Social/Professional Role and Identity’ (9), ‘Beliefs about Capabilities’ (8), ‘Optimism’ (4), ‘Beliefs about Consequences’ (5), ‘Reinforcement’ (7), ‘Intentions’ (3), ‘Goals’ (6), ‘Memory, Attention and Decision Processes’ (5), ‘Environmental Context and Resources’ (6), ‘Social Influences’ (11), ‘Emotions’ (7), and ‘Behavioural Regulation’ (3). The full version of the new framework is shown in Table 2.

**Table 2:** The refined framework based on results of the open and closed sort tasks

**Domain (definition**^**1**^**)**	**Constructs**
**1. Knowledge**	Knowledge (including knowledge of condition /scientific rationale)
(An awareness of the existence of something)	Procedural knowledge
	Knowledge of task environment
**2. Skills**	Skills
(An ability or proficiency acquired through practice)	Skills development
	Competence
	Ability
	Interpersonal skills
	Practice
	Skill assessment
**3. Social/Professional Role and Identity**	Professional identity
(A coherent set of behaviours and displayed personal qualities of an individual in a social or work setting)	Professional role
	Social identity
	Identity
	Professional boundaries
	Professional confidence
	Group identity
	Leadership
	Organisational commitment
**4. Beliefs about Capabilities**	Self-confidence
(Acceptance of the truth, reality, or validity about an ability, talent, or facility that a person can put to constructive use)	Perceived competence
	Self-efficacy
	Perceived behavioural control
	Beliefs
	Self-esteem
	Empowerment
	Professional confidence
**5. Optimism**	Optimism
(The confidence that things will happen for the best or that desired goals will be attained)	Pessimism
	Unrealistic optimism
	Identity
**6. Beliefs about Consequences**	Beliefs
(Acceptance of the truth, reality, or validity about outcomes of a behaviour in a given situation)	Outcome expectancies
	Characteristics of outcome expectancies
	Anticipated regret
	Consequents
**7. Reinforcement**	Rewards (proximal / distal, valued / not valued, probable / improbable)
(Increasing the probability of a response by arranging a dependent relationship, or contingency, between the response and a given stimulus)	Incentives
	Punishment
	Consequents
	Reinforcement
	Contingencies
	Sanctions
**8. Intentions**	Stability of intentions
(A conscious decision to perform a behaviour or a resolve to act in a certain way)	Stages of change model
	Transtheoretical model and stages of change
**9. Goals**	Goals (distal / proximal)
(Mental representations of outcomes or end states that an individual wants to achieve)	Goal priority
	Goal / target setting
	Goals (autonomous / controlled)
	Action planning
	Implementation intention
**10. Memory, Attention and Decision Processes**	Memory
(The ability to retain information, focus selectively on aspects of the environment and choose between two or more alternatives)	Attention
	Attention control
	Decision making
	Cognitive overload / tiredness
**11. Environmental Context and Resources**	Environmental stressors
(Any circumstance of a person's situation or environment that discourages or encourages the development of skills and abilities, independence, social competence, and adaptive behaviour)	Resources / material resources
	Organisational culture /climate
	Salient events / critical incidents
	Person x environment interaction
	Barriers and facilitators
**12. Social influences**	Social pressure
(Those interpersonal processes that can cause individuals to change their thoughts, feelings, or behaviours)	Social norms
	Group conformity
	Social comparisons
	Group norms
	Social support
	Power
	Intergroup conflict
	Alienation
	Group identity
	Modelling
**13. Emotion**	Fear
(A complex reaction pattern, involving experiential, behavioural, and physiological elements, by which the individual attempts to deal with a personally significant matter or event)	Anxiety
	Affect
	Stress
	Depression
	Positive / negative affect
	Burn-out
**14. Behavioural Regulation**	Self-monitoring
(Anything aimed at managing or changing objectively observed or measured actions)	Breaking habit
	Action planning

^1^All definitions are based on definitions from the American Psychological Associations’ Dictionary of Psychology [36].

## **Discussion**

This validation study, using open and closed sort tasks, has shown good support for the basic structure of the TDF and led to refinements producing 14 domains: ‘Knowledge’, ‘Skills’, ‘Social/Professional Role and Identity’, ‘Beliefs about Capabilities’, ‘Optimism’, ‘Beliefs about Consequences’, ‘Reinforcement’, ‘Intentions’, ‘Goals’, ‘Memory, Attention and Decision Processes’, ‘Environmental Context and Resources’, ‘Social Influences’, ‘Emotions’, and ‘Behavioural Regulation’. There are three key advantages of this framework. First, there is comprehensive coverage of possible influences on behavior. Second, there is clarity about each kind of influence, as a result of each domain being specified by component constructs. Third, the framework makes links between theories of behaviour change and techniques of behaviour change to address implementation problems. The framework can be applied by gathering either qualitative data (interviews or focus groups) or quantitative data (*e.g.*, by questionnaires). The findings have strengthened the evidence for the structure and content of the domains, increasing confidence in the usefulness of the TDF as an approach to assessing implementation and other behaviour problems, and laying the foundation for theoretically informed interventions.

To the authors’ knowledge, Fuzzy Cluster Analysis and Discriminant Content Validity have not been used in combination to determine the validity of a framework structure. By combining these methods, we have investigated the validity of the original framework both when the original domain labels were, and were not, presented. The results from both the open and closed sort tasks generally replicated the original framework, which adds confidence to the validity of the framework’s structure.

The study findings pointed to some changes in the framework, which had good face validity. First, there was a separation and clarification of a number of existing domains. The separation of ‘Motivation and Goals’ into two domains of ‘Intentions’ and ‘Goals’ was indicated by both the closed and open sort task results and was particularly apparent in the labels provided by the participants, with labels relating to ‘intentions’ and ‘goals’ amongst the most frequently used. The APA dictionary of psychology defines a goal as ‘the end state toward which a human or non-human animal is striving: the purpose of an activity or endeavour.’ [36] and defines intention as ‘a conscious decision to perform a behaviour; a resolve to act in a certain way or an impulse for purposeful action. In experiments, intention is often equated with goals defined by the task instructions.’ [36]. Therefore ‘Goals’ tends to refer to an end state that can be seen as a preferred outcome, whereas ‘Intentions’ is concerned with the resolve to initiate or terminate a behaviour. The separation of ‘Beliefs about Consequences’ into two domains, one retaining the original name and one termed ‘Reinforcement’, made psychological sense. The former refers to beliefs whereas the latter refers to constructs of associative learning. There was also a separation within the ‘Beliefs about Capabilities’ domain with a separate ‘Optimism’ domain being formed. This separation makes psychological sense in that the constructs in the optimism cluster concern general disposition rather than specific capabilities required to achieve an outcome. The domain ‘Behavioural Regulation’ is clearer in the refined framework where it refers to self-regulatory processes rather than including a mixture of self-regulation and goal-related constructs, as was the case in the original TDF.

Second, the ‘Nature of the Behaviours’ domain was dropped in the new framework, because its original component constructs were not assigned to the domain in the closed sort, and there was no cluster representing the ‘Nature of the Behaviours’ in the open sort. This strengthens the coherence of the new TDF because the domain did not sit easily in the original TDF. It was defined as the ‘Essential characteristics of the behaviour’, had constructs relating to habit and experiences/past behaviours, and constituted an outcome, or dependent variable, rather than an independent variable. Whilst understanding the nature of behaviours is absolutely key to analyzing implementation and other behavioural problems, analysing the nature of behaviour is a different task than analysing influences on behaviour. A complementary theoretical approach to analyzing behaviour as a basis for intervention design has been recently developed, as part of the ‘Behaviour Change Wheel’ [46]. Previous studies that have adopted the TDF framework have seldom used the ‘Nature of the Behaviours’ domain [17]. Furthermore, where the domain has been used, in relation to changing transfusion practice, it was noted that when participants were asked questions relating to the ‘Nature of the Behaviours’ domain they often repeated answers that were previously given in response to questions relating to the ‘Behavioural Regulation’ domain [19], therefore making responses in respect to ‘Nature of the Behaviours’ redundant. This along with empirical evidence shown in the present study shows a clear indication that the ‘Nature of the Behaviours’ domain should be considered differently to the components of the TDF.

In designing interventions, the TDF fits well with the Behaviour Change Wheel (BCW) [46] referred to above. The BCW characterises the target behavior in terms of Capability, Opportunity and Motivation (the COM-B system in the Behaviour Change Wheel), with Capability divided into psychological and physical capability, Opportunity divided into social and physical opportunity and Motivation divided into reflective and automatic motivation. The domains from the refined framework have been independently mapped onto the COM-B segments by three experts in behavior change, with 100% agreement (Table 3). Use of the COM-B may help identify the TDF domains that are likely to be important in changing behaviour. By starting with a behavioural analysis such as this, intervention designers can be selective about the domains they investigate to inform the nature of the intervention.

**Table 3:** Mapping of the Behaviour Change Wheel’s COM-B system to the TDF Domains

**COM-B component**		**TDF Domain**
Capability	Psychological	Knowledge
		Skills
		Memory, Attention and Decision Processes
		Behavioural Regulation
	Physical	Skills
Opportunity	Social	Social Influences
	Physical	Environmental Context and Resources
Motivation	Reflective	Social/Professional Role & Identity
		Beliefs about Capabilities
		Optimism
		Beliefs about Consequences
		Intentions
		Goals
	Automatic	Social/Professional Role & Identity
		Optimism
		Reinforcement
		Emotion

Research using the TDF has identified lack of knowledge as a potential barrier to a number of professional health behaviours, including hand hygiene [17], changing transfusion practice [19], and the adoption of tobacco use cessation counseling in dental practices [21]. However, for most health-related behaviours that are the target of theoretically-based behaviour change interventions (*e.g.*, smoking, healthy eating, physical activity), knowledge is not an important source of variance [47-52]. This may be why participants did not identify a separate domain for knowledge, but that it has been identified as an important influence on some health professional behaviours. We therefore recommend that knowledge be assessed along with the other TDF domains.

Of the original 112 unique constructs in the TDF, 34 have been removed. They appear to be a mixture of rather vague constructs (*e.g.*, Mindsets), very general constructs (*e.g.*, Review), ambiguous constructs (*e.g.*, Commitment), and infrequently used constructs in behaviour change theory (*e.g.*, Generating alternatives). Because constructs that are ‘poorly defined’, ‘undifferentiated’, and ‘imprecisely partitioned’ have previously been found to influence the content validity of assessment instruments [53], their exclusion from the refined framework seems warranted. The remaining constructs stand as a more defined, focused set of constructs that are more relevant to behaviour change theory and more precisely partitioned into domains. Within these remaining constructs, there are also a number of constructs that appear in more than one domain. Such allocations indicate the relevance of constructs across different domain contexts. For example, ‘Action Planning’ appears in both the ‘Goals’ domain and the ‘Behavioural Regulation’ domain and can be considered as being influential in achieving a particular goal (*e.g.* I plan to achieve goal x through specific actions) and also in regulating behaviour (*e.g.* in a certain situation I plan to behave in a particular way).

Two domains showed weak clustering: ‘Environmental Context and Resources’ and ‘Behavioural Regulation’. However, these domains, alongside the domain of ‘Knowledge’, were comprised of constructs consistently assigned to them when the original domain labels were presented in the closed sort task. This suggests that people are clear about the constructs within these domains when the domain labels are present. A second limitation is that the refined framework is limited to the constructs identified in the original framework. Whilst the current range of component constructs is quite extensive, it does not cover all theories of behaviour change [54], and future research is likely to identify others that are important to behaviour change. Just as the current framework is an advance on the 2005 version, so future work is likely to improve it further. The issue of how to evaluate appropriateness and quality of theories in given contexts is an under-researched area, but one that is beginning to be addressed [54].

## **Conclusions**

Through a three-step validation process, the present research has identified a refined version of the original TDF. This refined framework contains 14 domains and 84 component constructs. The strength of the framework validation stems from the methods used. Both the closed and open sort task methods alongside DCV and Fuzzy Cluster Analysis have provided complementary methods for examining the structure of the original framework. DCV methods assessed the confidence of allocation of constructs to the described domains, and the Fuzzy Cluster Analysis led to a refinement of the structure of the framework. The TDF has proved useful across a number of healthcare systems and this empirically-based refinement lays the basis for stronger explanatory and predictive power, and therefore increased usefulness in informing interventions to improve implementation and bring about other behaviour change.

## **Competing interests**

SM and DOC are both Associate Editors of Implementation Science.

## **Authors’ contributions**

JC conducted preparation of materials, data collection, data analysis, and drafted the manuscript. DOC and SM commented on and aided in the drafting of the manuscript. All authors read and approved the final manuscript.

## Supplementary Material

Additional file 1 **Additional file 1:** Constructs from the original Theoretical Domains Framework and associated definitions [55-68]**.** Click here for file

Additional file 2 **Additional file 2:** Instructions and additional questions given to participants. Instructions, consent information and additional questions given to participants. Click here for file

Additional file 3 **Additional file 3:** Mantel correlation values by participant. Mantel correlation coefficients for each participant. Click here for file

Additional file 4 **Additional file 4:** Comparison of the refined framework, closed sort task, and open sort task groupings with related mean confidence ratings, Intra-Class Correlation (ICC) values and silhouette values. Click here for file

Additional file 5 **Additional file 5:** Membership values (%) for each construct to each domain cluster defined in the cluster analysis. Click here for file
