# Supplementary material for: Validation of the theoretical domains framework for use in behaviour change and implementation research
Source: Implement Sci. 2012 Apr 24;7:37. doi: 10.1186/1748-5908-7-37 (PMC3483008; doi:10.1186/1748-5908-7-37)
Supplement: Additional file 1 — Constructs from the original Theoretical Domains Framework and associated definitions [55-68]. [file 1748-5908-7-37-S1.pdf]

Additional file 1. Constructs from the original Theoretical Domains Framework and associated definitions (superscript number refers to definition source)

Construct	Definition
**Ability**	Competence or capacity to perform a physical or mental act. Ability may be either unlearned or acquired by education and practice.[2]
**Action planning**	The action or process of forming a plan regarding a thing to be done or a deed.[7]
**Affect**	An experience or feeling of emotion, ranging from suffering to elation, from the simplest to the most complex sensations of feelings, and from the most normal to the most pathological emotional reactions.[1]
**Alienation**	Estrangement from one's social group; a deep seated sense of dissatisfaction with one's personal experiences that can be a source of lack of trust in one's social or physical environment or in oneself; the experience of separation between thoughts and feelings.[1]
**Anticipated regret**	A sense of the potential negative consequences of a decision that influences the choice made: for example an individual may decide not to make an investment because of the feelings associated with an imagined loss.[1]
**Anxiety**	A mood state characterised by apprehension and somatic symptoms of tension in which an individual anticipates impending danger, catastrophe or misfortune.[1]
**Appraisal**	The cognitive evaluation of a phenomenon or event. In theories of emotions, cognitive appraisals are seen as determinants of emotional experience.[1]
**Attention**	A state of awareness in which the senses are focussed selectively on aspects of the environment and the central nervous system is in a state of readiness to respond to stimuli.[1]
**Attention control**	The extent to which a person can concentrate on relevant cues and ignore all irrelevant cues in a given situation.[2]
**Attitudes**	Any subjective belief or evaluation associated with an object or behaviour.[2]
**Barriers and facilitators**	In psychological contexts barriers/facilitators are mental, emotional or behavioural limitations/strengths in individuals or groups.[1]
**Beliefs**	The thing believed; the proposition or set of propositions held true.[6]
**Breaking habit**	To discontinue a behaviour or sequence of behaviours that is automatically activated by relevant situational cues.[2]
**Burn-out**	Physical, emotional or mental exhaustion, especially in one's job or career, accompanied by decreased motivation, lowered performance and negative attitudes towards oneself and others.[1]
**Certainty of intentions**	Assuredness of one's resolve to act in a certain way. [7]
**Champions/To champion**	To fight for another or for a cause.[15]
**Change Management**	A process during which the changes to a system are implemented in a controlled manner by following a pre-defined framework.

Construct	Definition
**Characteristics of outcome expectancies**	Characteristics of the cognitive, emotional and behavioural outcomes that individuals believe are associated with future or intended behaviours and that are believed to either promote or inhibit these behaviours. These include whether they are sanctions/rewards, proximal/distal, valued/not valued, probable/improbable, salient/not salient, perceived risks or threats.[2]
**Cognitive overload/tiredness**	The situation in which the demands placed on a person by mental work are greater than a person's mental abilities.[1]
**Commitment**	The act of binding yourself (intellectually or emotionally) to a course of action.[5]
**Competence**	One's repertoire of skills, and ability especially as it is applied to a task or set of tasks.[2]
**Conflict - competing demands, conflicting roles**	The actual or perceived incompatibility between the performance of two or more behaviours.[2]
**Consequents**	An outcome of behaviour in a given situation.[1]
**Contingencies**	A conditional probabilistic relation between two events. Contingencies may be arranged via dependencies or they may emerge by accident.[1]
**Control of behaviour, material and social environment**	Authority, power or influence over events, behaviours, situations or people. [2]
**Coping strategies**	An action, series of actions, or a thought process used in an attempt to reduce stress or used to modify one's reaction to a stressful/unpleasant situation. Coping strategies typically involve a conscious and direct approach to problems.[2]
**Crew resource management**	A management system that makes optimum use of all available resources, equipment, procedures and people.[8]
**Decision making**	The cognitive process of choosing between two or more alternatives, ranging from the relatively clear cut to the complex.[1]
**Depression**	A mental state that presents with depressed mood, loss of interest or pleasure, feelings of guilt or low self-worth, disturbed sleep or appetite, low energy, and poor concentration.[11]
**Direct experience**	The experience gained through immediate sense perception.[8]
**Empowerment**	The promotion of the skills, knowledge and confidence necessary to take great control of one's life as in certain educational or social schemes; the delegation of increased decision-making powers to individuals or groups in a society or organisation.[1]
**Environmental stressors**	External factors in the environment that cause stress.[2]
**Evaluation**	A careful examination or overall appraisal of something to determine its worth, value or desirability; a determination of the success of something in achieving defined goals; the interpretation of test results and experimental data.[1]
**Fear**	An intense emotion aroused by the detection of imminent threat, involving an immediate alarm reaction that mobilises the organism by triggering a set of physiological changes.[1]
**Feedback**	The return of information about progress on or the outcome of a process or activity.[16]

Construct	Definition
**Generating alternatives**	Thinking of other ways of dealing with a situation or problem.[2]
**Goal priority**	Order of importance or urgency of end states toward which one is striving.[2]
**Goal/target setting**	A process that establishes specific time based behaviour targets that are measurable, achievable and realistic.[1]
**Goals (autonomous/controlled)**	The end state toward which one is striving: the purpose of an activity or endeavour. It can be identified by observing that a person ceases or changes its behaviour upon attaining this state; proficiency in a task to be achieved within a set period of time.[2]
**Goals (distal/proximal)**	Desired state of affairs of a person or system, these may be closer (proximal) or further away (distal).[8]
**Group conformity**	The act of consciously maintaining a certain degree of similarity to those in your general social circles.[8]
**Group identity**	The set of behavioural or personal characteristics by which an individual is recognizable [and portrays] as a member of a group.[8]
**Group norms**	Any behaviour, belief, attitude or emotional reaction held to be correct or acceptable by a given group in society.[7]
**Hierarchy**	A clear order of individuals on some behavioural dimension such as dominance-submission.[1]
**Identity**	An individual's sense of self defined by a) a set of physical and psychological characteristics that is not wholly shared with any other person and b) a range of social and interpersonal affiliations (e.g., ethnicity) and social roles.[1]
**Illness representations**	Organised beliefs of illness acquired through the media, personal experience and from family and friends' experiences, beliefs, descriptions and knowledge of particular disorders.[3]
**Implementation intention**	The plan that one creates in advance of when, where and how one will enact a behaviour.[8]
**Incentives**	An external stimulus, such as condition or object, that enhances or serves as a motive for behaviour.[1]
**Intention**	A conscious decision to perform a behaviour; a resolve to act in a certain way or an impulse for purposeful action. In experiments, intention is often equated with goals defined by the task instruction.[1]
**Intergroup conflict**	Disagreement or confrontation between two or more groups and their members. This may involve physical violence, interpersonal discord, or psychological tension.[13]
**Interpersonal skills**	An aptitude enabling a person to carry on effective relationships with others, such as an ability to cooperate, to assume appropriate social responsibilities or to exhibit adequate flexibility.[1]
**Intrinsic motivation**	An incentive to engage in a specific activity that derives from the activity itself rather than because of any external benefits that might be obtained.[1]
**Knowledge**	An awareness of the existence of something.[1]
**Knowledge of task environment**	Knowledge of the social and material context in which a task is undertaken.[2]

Construct	Definition
**Leadership**	The processes involved in leading others, including organising, directing, coordinating and motivating their efforts toward achievement of certain group or organisation goals.[1]
**Learning**	The process of acquiring new and relatively enduring information, behaviour patterns or abilities, characterised by modification of behaviour as a result of practice, study or experience.[1,5]
**Management commitment**	The binding of a governing body of an organization or business to a course of action.[7]
**Memory**	The ability to retain information or a representation of a past experience, based on the mental processes of learning or encoding retention across some interval of time, and retrieval or reactivation of the memory; specific information of a specific past.[1]
**Mindset**	An established set of attitudes regarded as typical of a particular group's social or cultural values; the outlook, philosophy, or values of a person; frame of mind, attitude, disposition.[6]
**Modelling**	In developmental psychology the process in which one or more individuals or other entities serve as examples (models) that a child will copy.[1]
**Moderators of the intention behaviour gap**	Factors that affect the relationship between what one intends to do and what one actually does.
**Negotiation**	A reciprocal communication process in which two or more parties to a dispute examine specific issues, explain their positions and exchange offers and counter-offers in an attempt to identify a solution or outcome that is acceptable to all parties.[1]
**Optimism**	The attitude that outcomes will be positive and that people's wishes or aims will ultimately be fulfilled.[2]
**Organisational commitment**	An employee's dedication to an organisation and wish to remain part of it. Organisational commitment is often described as having both an emotional or moral element and a more prudent element.[1]
**Organisational culture/climate**	A distinctive pattern of thought and behaviour shared by members of the same organisation and reflected in their language, values, attitudes, beliefs and customs.[1]
**Organisational development**	The application of principles and practices drawn from psychology, sociology and related fields to the planned improvement of organisational effectiveness.[1]
**Outcome expectancies**	Cognitive, emotional, behavioural, and affective outcomes that are assumed to be associated with future or intended behaviours. These assumed outcomes can either promote or inhibit future behaviours.[2]
**Past behaviour**	Previous manner of conducting oneself.[7]
**Perceived behavioural control**	An individual's perception of the ease or difficulty of performing the behaviour of interest.[9]
**Perceived competence**	An individual's belief in his or her ability to learn and execute skills.[1]
**Person x environment interaction**	Interplay between the individual and their surroundings.

Construct	Definition
**Pessimism**	The attitude that things will go wrong and that people's wishes or aims are unlikely to be fulfilled.[1]
**Positive/negative affect**	The internal feeling/state that occurs when a goal has/has not been attained, a source of threat has/has not been avoided, or the individual is/is not satisfied with the present state of affairs.[1]
**Power**	The capacity to influence others, even when they try to resist this influence.[1]
**Practice**	Repetition of an act, behaviour, or series of activities, often to improve performance or acquire a skill.[1]
**Procedural knowledge**	Knowing how to do something.[4]
**Professional boundaries**	The bounds or limits relating to, or connected with a particular profession or calling.[7]
**Professional confidence**	An individual's belief in his or her repertoire of skills, and ability especially as it is applied to a task or set of tasks.[7]
**Professional identity**	The characteristics by which an individual is recognised relating to, connected with or befitting a particular profession.[7]
**Professional role**	The behaviour considered appropriate for a particular kind of work or social position.[7]
**Project management**	Defining and achieving targets while optimizing the use of resources over the course of a project.[8]
**Punishment**	The process in which the relationship between a response and some stimulus or circumstance results in the response becoming less probable; a painful, unwanted or undesired event or circumstance imposed as a penalty on a wrongdoer.[1]
**Reinforcement**	A process in which the frequency of a response is increased by a dependent relationship or contingency with a stimulus.[2]
**Representation of tasks**	A mental model of goal-directed activities.[2]
**Resources/material resources**	Commodities and human resources used in enacting a behaviour.[2]
**Review**	To look over or through in order to correct or improve; to revise.[6]
**Rewards (proximal/distal, valued/not valued, probable/improbable)**	Return or recompense made to, or received by a person contingent on some performance.[7]
**Routine/automatic/habit**	A well-learned behaviour or automatic sequence of behaviours that is relatively situation specific. At its extreme the behaviour has become a reflex; independent of motivation, cognitive influence or conscious control.[2]
**Salient events/critical incidents**	Occurrences that one judges to be distinctive, prominent or otherwise significant.[2]
**Sanctions**	A punishment or other coercive measure, usually administered by a recognised authority, that is used to penalise and deter inappropriate or unauthorised actions.[1]
**Schemas**	A collection of basic knowledge about a concept or entity that serves as a guide to perception, interpretation, imagination or problem solving.[1]
**Self-confidence**	Self-assurance or trust in one's own abilities, capabilities and judgment.[1]

Construct	Definition
**Self-efficacy**	An individual's capacity to act effectively to bring about desired results, as perceived by the individual.[2]
**Self-esteem**	The degree to which the qualities and characteristics contained in one's self- concept are perceived to be positive.[1]
**Self-monitoring**	A method used in behavioural management in which individuals keep a record of their behaviour, especially in connection with efforts to change or regulate the self; a personality trait reflecting an ability to modify one's behaviour in response to situation.[1]
**Sensitisation**	A form of non-associative learning in which an organism becomes more responsive to most stimuli after being exposed to unusually strong or painful stimuli; the increased effectiveness of an eliciting stimulus as function of its presentation.[1]
**Skill assessment**	A judgment of the quality, worth, importance, level, or value of an ability or proficiency acquired through training and practice.[2]
**Skills**	An ability or proficiency acquired through training and/or practice.[2]
**Skills development**	The gradual acquisition or advancement through progressive stages of an ability or proficiency acquired through training and practice.[2, 7]
**Social comparisons**	The process by which people evaluate their attitudes, abilities, or performance relative to others.[14]
**Social identity**	The set of behavioural or personal characteristics by which an individual is recognizable [and portrays] as a member of a social group.[1]
**Social norms**	Socially determined consensual standards that indicate a) what behaviours are considered typical in a given context and b) what behaviours are considered proper in the context.[1]
**Social pressure**	The exertion of influence on a person or group by another person or group.[1]
**Social support**	The apperception or provision of assistance or comfort to others, typically in order to help them cope with a variety of biological, psychological and social stressors. Support may arise from any interpersonal relationship in an individual's social network, involving friends, neighbours, religious institutions, colleagues, caregivers or support groups.[2]
**Stability of intentions**	Ability of one's resolve to remain in spite of disturbing influences.[7]
**Stages of Change model**	A model that proposes that behaviour change is accomplished through five specific stages: Pre-contemplation, Contemplation, Preparation, Action, and Maintenance.[17]
**Stress**	A state of physiological or psychological response to internal or external stressors.[1]
**Supervision**	Management by overseeing the performance or operation of a person of group.[12]
**Team working**	Cooperative effort toward a common goal or on a common project.[1]
**Threat**	A condition that is appraised as a danger to oneself or well-being or to a group.[1]

Construct	Definition
**Transtheoretical model and stages of change**	A five-stage theory to explain changes in people's health behaviour. It suggests that change takes time, that different interventions are effective at different stages, and that there are multiple outcomes occurring across the stages.[1]
**Unrealistic optimism**	The inert tendency for humans to over-rate their own abilities and chances of positive outcomes compared to those of other people.[10]

N.B. Definitions for the original Theoretical Domains Framework domains are available from the first author on request.

Definition source (construct labels were used as keywords in each of the definition searches; see reference section for full citations): [1] APA Dictionary of Psychology [36], [2] Based on definition(s) from APA Dictionary of Psychology [36], [3] Based on definition from Taylor (2003) [55], [4] The Macmillan Dictionary of Psychology (2nd Edition) [56], [5] Based on definition from WordNet [57], [6] Oxford English Dictionary Online [58], [7] Based on definition(s) from Oxford English Dictionary Online [58], [8] Based on definition from En.wikipedia.org/wiki entry [59], [9] Based on definition from Azjen (1991) [60], [10] Based on definition from Ogden (2000) [61], [11] World Health Organisation (WHO) [62], [12] Dictionary.com [63], [13] Forsythe (2009) [64], [14] Based on definition from Alicke (2000) [65], [15] YourDictionary.com [66], [16] The Free Dictionary [67], [17] Based on definition from Proschaska and DiClemente (1984) [68].
