# Supplementary material for: Validation of the theoretical domains framework for use in behaviour change and implementation research
Source: Implement Sci. 2012 Apr 24;7:37. doi: 10.1186/1748-5908-7-37 (PMC3483008; doi:10.1186/1748-5908-7-37)
Supplement: Additional file 2 — Instructions and additional questions given to participants. Instructions, consent information and additional questions given to participants. [file 1748-5908-7-37-S2.pdf]

Additional file 2. Instructions and additional questions given to participants

Initial study introduction and consent (both sort tasks)

Before you decide whether to take part please read the following information carefully. Please contact James Cane (j.cane@ucl.ac.uk), the lead researcher on this project, if there is anything that is not clear.

The aim of this study is to examine the extent to which constructs are grouped together by those who have expertise in behaviour change theory. The construct groups provided through this task will be compared with a developed theoretical framework of behaviour change. Through this process we will be able to validate the framework, and also highlight any areas for improvement. Our pilot has suggested that this task takes around an hour to complete.

To take part, you must have been involved in research, teaching or practice relating to behaviour change theory AND be unaware of the theoretical framework reported by Michie, S., Johnston, M., Abraham, C., Lawton, R., Parker, D., Walker A. (on behalf of the Psychological Theory Group)(2005) other than through information provided in the recruitment emails for this project.

If you decide to take part you will only be asked for minimal personal information (age, sex, country of residence, and information about your experience with behaviour change theory / interventions). All data will be treated with full confidentiality. You can withdraw from the study at any point and without reason. The data you provide will be related to a unique personal identifier (generated on task completion) that you can use if you would like your data to be removed. By ticking the following check-box you give your consent to take part in the study.

Initial Questions (both sort tasks)

1.) Country of residence : [ ] 2.) Age : [ ] 3.) Sex : ○ Male ○ Female

4.) Other than information provided in the recruitment emails for this study, do you have knowledge of the theoretical framework reported by Michie, S., Johnston, M., Lawton, R., Parker, D., Walker A. (on behalf of the Psychological Theory Group)(2005) or have you ever conducted research / teaching using this theoretical framework?

○ Yes ○ No

5.) How long (in years / months) have you been working with behaviour change theories (conducting research, teaching, studying)?

Years : [ ] Months : [ ]

6.) In what capacity have you been working with, or had knowledge of, behaviour change theories (in research, teaching, studying, etc.)?

[ ]

How would you rate your expertise (i.e. knowledge and familiarity) in the following areas?

	A great deal	Quite a bit	Some	A little	None
Delivering behaviour change interventions	○	○	○	○	○
Behaviour change theory	○	○	○	○	○

Instructions

Closed Sort Task

It is important you read through these instructions prior to completing the sorting task on the next page.

Task Overview :
Your task is to sort construct items into category boxes based on how relevant you think they are to the categories. You will also rate your confidence that you think a construct item belongs to a category. There are 112 construct items to assign in total and there is no time limit to complete this task.

How to assign a construct to a category box:

1. Choose a Category

Choose which category you would like the construct item to go into by using the *Category* drop down menu to the right of the construct.

2. Choose a Confidence Rating

It is very important that you provide a confidence rating for each of the constructs you have assigned. Rate how confident you are that the construct belongs to the category by using the *Confidence* drop down menu. Confidence ratings go from 1 - not confident at all to 10 - extremely confident.

3. Click the *select* button

This moves the construct item into your selected category box with the confidence rating next to it.

4. Choose another category and confidence rating (optional)

If you think the construct belongs to more than one category choose another category and confidence rating, press the *select* button again. Your item should then appear in more than one category box.

5. Click the *next* button

Once you are happy with your selection(s) click on the *next* button to move onto the next construct item to assign.

Definitions

To get a definition of the construct item you are sorting simply roll-over the construct item in the ITEM box and a definition will appear. These definitions may help you when sorting items.

Reviewing selections and reallocating items:

Once you have allocated all of the constructs you should take time to review your choices.

If you would like to reallocate a construct, or re-rate the confidence value, **double click** on the construct (using the left mouse button) and it will appear in the item box at the top of the screen again. You can then allocate the construct to a different group or give it a different rating.

Restrictions on allocating items:

You can allocate a construct item to multiple categories, but you cannot allocate the same construct to the same category more than once (a warning is given if you try to do this).

Submitting your allocations

Once you are happy with your allocations you can click on the ***continue*** button to submit them.

Downloading Instructions

If you would like to refer to these instructions during the task click on 'download instructions' at the bottom of this screen (this will open the instructions in a word document that can be printed - choose 'HTML document' if a dialogue box appears). A list of items you will be sorting can also be printed by using the 'Download Items' Button. Please note the items in the print version will not be in the same order as shown in the task.

Instructions Open Sort

It is important you read through all of these instructions prior to completing the sorting task.

The task:

On the following screen you will see 24 empty category boxes and at the top of the screen you will see a construct item. Your task is sort the construct items into groups based on their semantic similarity. You can create as many groups as you feel are necessary up to a maximum of 24 (we suggest around 8 to 14 groups). You can allocate a construct to multiple groups. There are 112 constructs to assign to groups and there is no time limit to complete this task.

How to assign a construct item to a category box:

1. Choose a Category

Choose which category you would like the construct item to go into by using the *Category* drop down menu. To get started, you may want to put the first construct item into category 1 and then compare subsequent construct items with this allocation (i.e. compare whether construct 2 should be in the same group or a different group as construct 1 and so on).

2. Choose a Confidence Rating

It is very important that you provide a confidence rating for each of the constructs you are sorting. Rate how confident you are that the construct belongs to the category by using the ‘Confidence’ drop down menu. Confidence ratings go from 1 - not confident at all to 10 - extremely confident.

3. Click the *select* button

This moves the construct item into your selected category box with the confidence rating next to it.

4. Choose another category and confidence rating (optional)

If you think the construct belongs to more than one category choose another category and confidence rating, press the ***select*** button again. Your item will then appear in more than one category box.

5. Click the *next* button

Once you are happy with your selection(s) click on the *next* button to move onto the next construct item to assign.

6. Label your categories

PLEASE PROVIDE A LABEL FOR ALL CATEGORIES THAT YOU CREATE. To label the categories simply move the mouse cursor to the label box and type in a label. You can also type in a description of the group in the description box.

Definitions

To get a definition of the construct item you are sorting simply roll-over the construct item in the ITEM box and a definition will appear. These definitions may help you when sorting items.

Reviewing selections and reallocating items:

Once you have allocated all of the constructs you should take time to review your choices.

If you would like to reallocate a construct, or re-rate the confidence value, double click on the construct (using the left mouse button) and it will appear in the item box at the top of the screen again. You can then allocate the construct to a different group or give it a different rating.

Submitting your allocations

Once you are happy with your allocations you can click on the ***continue*** button to submit them. The data may take a few seconds to be submitted.

Downloading Instructions

If you would like to refer to these instructions during the task click on 'download instructions' at the bottom of this screen (this will open the instructions in a word document that can be printed - choose 'HTML document' if a dialogue box appears). A list of items you will be sorting can also be printed by using the 'Download Items' Button. Please note the items in the print version will not be in the same order as shown in the task.
