# Supplementary material for: Validation of the theoretical domains framework for use in behaviour change and implementation research
Source: Implement Sci. 2012 Apr 24;7:37. doi: 10.1186/1748-5908-7-37 (PMC3483008; doi:10.1186/1748-5908-7-37)
Supplement: Additional file 3 — Mantel correlation values by participant. Mantel correlation coefficients for each participant. [file 1748-5908-7-37-S3.pdf]

Additional File 3. Mantel correlation values by participant

Participant	Mantel correlation
1	0.18
2	0.16
3	0.13
4	0.16
5	0.13
6	0.13
7	0.23
8	0.21
9	0.24
10	0.17
11	0.16
12	0.17
13	0.15
14	0.21
15	0.17
16	0.22
17	0.17
18	0.19
19	0.14

Values based on 1,000 permutations, all corrected p-values <0.02.
