# Supplementary material for: Validation of the theoretical domains framework for use in behaviour change and implementation research
Source: Implement Sci. 2012 Apr 24;7:37. doi: 10.1186/1748-5908-7-37 (PMC3483008; doi:10.1186/1748-5908-7-37)
Supplement: Additional file 4 — Comparison of the refined framework, closed sort task, and open sort task groupings with related mean confidence ratings, Intra-Class Correlation (ICC) values and silhouette values. [file 1748-5908-7-37-S4.pdf]

Additional File 4. Comparison of the refined framework, closed sort task, and open sort task groupings

**Original Framework Domains + related ICCs and congruency**		**Closed Sort Task**		**Open Sort Task**		
	**Refined framework domain name and constructs**	**Constructs achieving p<.05[a] (in order of confidence rating high – low)**	**Mean Confidence rating (95% Conf. Int. Lower, Upper)**	**Construct clusters (in order of s(i) values – decreasing; Italicised constructs silhouette value <.25)**	**Silhouette values s(i)**	**Av. *s(i)* of cluster *s(i)***
**1. Knowledge**	**1. Knowledge**					
ICC(C,1) = 0.39	Knowledge[b]	Knowledge[b]	8.50*** (6.91, 10.00)	- No cluster representing Knowledge -	-	-
CS Cong. = 40%	Procedural knowledge	Procedural knowledge	6.72*** (4.79, 8.65)			
OS Cong. = 0%	Knowledge of task environment	Knowledge of task environment	6.33** (4.22, 8.44)			
**2. Skills**	**2. Skills**					
ICC(C,1) = 0.38	Skills	Skills	8.56*** (7.24, 9.87)	Competence	0.59	-
CS Cong. = 88%	Skills development	Skills development	7.11*** (5.34, 8.88)	Skills	0.56	*0.45*
OS Cong. = 75%	Competence	Competence	6.39*** (4.49, 8.29)	Skill assessment	0.48	
	Ability	Ability	6.33*** (4.43, 8.23)	Ability	0.48	
	Interpersonal skills	Interpersonal skills	5.61** (3.47, 7.76)	Interpersonal skills	0.39	
	Practice	Practice	5.56** (3.41, 7.70)	Skills development	0.38	
	Skill assessment	Skill assessment	4.39* (2.31, 6.46)	Procedural knowledge	0.25	
**3. Social/ Professional Role and Identity**	**3. Social/ Professional Role and Identity**					
ICC(C,1) = 0.34	Professional identity	Professional identity	8.06*** (6.45, 9.66)	Organisational development	0.50	0.38
CS Cong. = 70%	Professional role	Professional role	7.94*** (6.38, 9.51)	Organisational culture/climate	0.45	*0.31*
OS Cong. = 40%	Social identity	Social identity	6.17** (4.09, 8.24)	Management commitment	0.41	
	Identity	Identity	6.06** (4.01, 8.1)	Professional role	0.40	
	Professional boundaries	Professional boundaries	6.00*** (4.19, 7.81)	Crew resource management	0.38	
	Professional confidence	Professional confidence	6.00*** (4.00, 8.00)	Leadership	0.37	
	Group identity	Leadership	5.33** (3.52, 7.15)	Change management	0.36	
	Leadership	Group identity	5.17** (3.15, 7.18)	Professional boundaries	0.36	
	Organisational commitment	Organisational commitment	4.39** (2.74, 6.04)	Organisational commitment	0.35	
				Supervision	0.34	
				Professional identity	0.34	
				Project management	0.31	
				Champions / To champion	*0.19*	
				Team working	*0.13*	
				Power	*0.02*	
				Hierarchy	*-0.03*	
**4. Beliefs about Capabilities**	**4. Beliefs about Capabilities**					
ICC(C,1) = 0.40	Self-confidence	Self-confidence	8.06*** (6.84, 9.27)	Self-efficacy	0.59	0.50
CS Cong. = 70%	Perceived competence	Perceived competence	7.78*** (6.23, 9.33)	Perceived competence	0.56	*0.45*

OS Cong. (Beliefs about Capabilities cluster) = 60%	Self-efficacy	Self-efficacy	6.94*** (4.94, 8.95)	Self-confidence	0.55	
	Perceived behavioural control	Perceived behavioural control	6.89*** (5.06, 8.72)	Perceived behavioural control	0.51	
	Beliefs	Self-esteem	6.50*** (4.85, 8.15)	Professional confidence	0.27	
	Self-esteem	Beliefs	6.06*** (4.21, 7.9)	*Self-esteem*	*0.20*	
	Empowerment	Empowerment	4.61* (2.45, 6.78)			
	Professional confidence	Professional confidence	4.39* (2.11, 6.67)			
OS Cong. (Optimism cluster) = 20%	**5. Optimism**					
	Optimism			Optimism	0.51	0.38
	Pessimism			Pessimism	0.42	*0.31*
	Unrealistic optimism			Unrealistic optimism	0.35	
	Identity			Identity	0.25	
				Mindsets	*-0.01*	
**5. Beliefs about Consequences**	**6. Beliefs about Consequences**					
ICC(C,1) = 0.25	Outcome expectancies	Outcome expectancies	7.61*** (6.06, 9.17)	Beliefs	0.61	0.55
CS Cong. = 26%	Chars. of outcome expectancies [b]	Chars. of outcome expectancies [b]	6.17*** (4.14, 8.19)	Attitudes	0.58	*0.48*
OS Cong. (Beliefs about Consequences cluster) = 21%	Beliefs	Beliefs	5.11*** (2.93, 7.29)	Outcome expectancies	0.56	
	Consequents	Consequents	4.67* (2.34, 6.44)	Chars. of outcome expectancies [b]	0.43	
	Anticipated regret	Anticipated regret	4.39* (2.44, 6.89)	*Illness representations*	*0.22*	
OS Cong. (Reinforcement cluster = 38%	**7. Reinforcement**					
	Rewards[b]			Rewards[b]	0.68	-
	Incentives			Incentives	0.64	*0.54*
	Punishment			Punishment	0.60	
	Sanctions			Sanctions	0.51	
	Contingencies			Contingencies	0.46	
	Reinforcement			Reinforcement	0.45	
	Consequents			Consequents	0.42	
**6. Motivation and Goals**	**8. Intentions**					
ICC(C,1) = 0.38	Stability of intentions	Goals (autonomous, controlled)	7.83*** (6.28, 9.39)	Stability of intentions	0.27	0.26
CS Cong. = 80%	Stages of change model	Intrinsic motivation	7.78*** (6.21, 9.35)	Stages of change model	0.26	*0.17*
OS Cong. (Intentions cluster) = 60%	Trans. model and stages of change[b]	Goal target /setting	7.67*** (6.21, 9.12)	Trans. model and stages of change[b]	0.25	
		Distal and proximal goals	7.33*** (5.86, 8.81)	*Certainty of intentions*	*0.23*	
		Goal priority	6.39*** (4.33, 8.45)	*Intention*	*0.18*	
		Intention	5.72** (3.57, 7.88)	*Commitment*	*0.13*	
		Stability of intentions	4.89* (2.73, 7.05)	*Intrinsic motivation*	*0.12*	
		Certainty of intentions	4.56* (2.58, 6.53)	*Mods. of the intention-behaviour gap*[b]	*-0.06*	
OS Cong. (Goals cluster) = 40%	**9. Goals**					
	Goals (distal / proximal)			Goal target/ setting	0.54	0.40
	Goal priority			Goals (distal / proximal)	0.44	*0.38*
	Goal / target setting			Goal priority	0.44	

	Goals (autonomous / controlled)			Goals (autonomous / controlled)	0.39	
	Action planning			Action planning	0.35	
	Implementation intention			Implementation intention	0.26	
	Goals (distal / proximal)			*Representation of tasks*	*0.22*	
**7. Memory, Attention and Decision Processes**	**10. Memory, attention and decision processes**					
ICC(C,1) = 0.31	Memory	Memory	8.33*** (6.70, 9.97)	Memory	0.25	0.25
CS Cong. = 100%	Attention	Attention	7.89*** (6.36, 9.41)	*Attention control*	*0.24*	*0.06*
OS Cong. = 100%	Attention control	Attention control	7.06*** (5.30, 8.81)	*Attention*	*0.23*	
	Decision making	Decision making	6.72*** (4.79, 8.65)	*Decision making*	*0.03*	
	Cognitive overload / tiredness	Cognitive overload / tiredness	3.50* (1.74, 5.26)	*Appraisal*	*0.01*	
				Schemas	*-0.12*	
				Cognitive overload / tiredness	*-0.20*	
**8. Environmental Context and Resources**	**11. Environmental Context and Resources**					
ICC(C,1) = 0.26	Environmental stressors	Environmental stressors	7.94*** (6.74, 9.14)	*Conflict- comp. demands, conf. roles*[b]	*0.03*	-
CS Cong. = 50%	Resources/material resources	Resources/material resources	6.61*** (4.66, 8.56)	*Barriers and facilitators*	*0.02*	*-0.12*
OS Cong. = 75%	Barriers and facilitators	Barriers and facilitators	5.11** (3.20, 7.02)	*Environmental stressors*	*-0.02*	
	Organisational culture/climate	Organisational culture climate	5.06** (3.08, 7.03)	*Salient events / critical incidents*	*-0.05*	
	Person x environment interaction	Person x environment interaction	4.72** (2.77, 6.67)	*Resources / material resources*	*-0.05*	
	Salient events/critical incidents	Salient events / critical incidents	4.61** (2.79, 6.43)	*Coping strategies*	*-0.10*	
				Knowledge of task environment	*-0.13*	
				Person x environment interaction	*-0.13*	
				Control of behaviour, material and social environment	*-0.14*	
				Knowledge	*-0.14*	
				Empowerment	*-0.19*	
				Negotiation	*-0.20*	
				Anticipated regret	*-0.21*	
				Threat	*-0.22*	
				Past Behaviour	*-0.22*	
**9. Social Influences**	**12. Social Influences**					
ICC(C,1) = 0.38	Social pressure	Social pressure	8.89*** (8.38, 9.4)	Group norms	0.71	-
CS Cong. = 27%	Social norms	Social norms	7.61*** (6.04, 9.18)	Group conformity	0.68	*0.62*
OS Cong. = 27%	Group conformity	Group conformity	7.39*** (6.34, 8.44)	Group identity	0.67	
	Social comparisons	Social comparisons	7.22*** (6.10, 8.35)	Social pressure	0.67	
	Group norms	Group norms	7.17*** (5.39, 8.94)	Social norms	0.64	
	Social support	Social support	7.06*** (5.03, 9.08)	Social support	0.63	
	Intergroup conflict	Intergroup conflict	5.61*** (3.85, 7.37)	Alienation	0.62	
	Power	Power	5.00** (3.08, 6.92)	Social comparisons	0.58	
	Group identity	Group identity	4.89** (2.84, 6.93)	Intergroup conflict	0.47	

	Alienation	Alienation	4.56** (2.56, 6.55)	Social identity	0.45	
	Modelling	Modelling	4.44* (2.34, 6.55)			
**10. Emotion**	**13. Emotion**					
ICC(C,1) = 0.45	Anxiety	Anxiety	8.33*** (7.05, 9.62)	Anxiety	0.69	-
CS Cong. = 77%	Fear	Fear	8.17*** (6.64, 9.69)	Depression	0.67	*0.59*
OS Cong. = 77%	Affect	Affect	8.17*** (6.62, 9.71)	Positive / negative affect	0.57	
	Stress	Stress	7.22*** (5.47, 8.97)	Stress	0.57	
	Depression	Depression	7.11*** (5.31, 8.91)	Fear	0.56	
	Positive / negative affect	Positive / negative affect	6.44*** (4.31, 8.58)	Affect	0.54	
	Burn-out	Burn-out	5.72** (3.69, 7.75)	Burn-out	0.52	
**11. Behavioural Regulation**	**14. Behavioural Regulation**					
ICC(C,1) = 0.14	Self-monitoring	Self monitoring	6.89*** (5.20, 8.58)	*Learning*	*0.22*	-
CS Cong. = 20%	Breaking habit	Breaking habit	5.78*** (4.08, 7.47)	*Review*	*0.18*	*0.06*
OS Cong. = 20%	Action planning	Action planning	4.94*** (2.82, 7.06)	*Breaking habit*	*0.16*	
				Direct experience	*0.16*	
				Self-monitoring	*0.15*	
				Evaluation	*0.06*	
				Generating alternatives	*0.05*	
				Feedback	*0.04*	
				Practice	*-0.04*	
				Sensitisation	*-0.05*	
				Routine / automatic / habit	*-0.06*	
				Modelling	*-0.13*	
**12. Nature of the Behaviours**<br>ICC(C,1) = 0.07<br>CS Cong. = 20%		Routine / automatic / habit	4.89** (2.82, 6.96)			

Constructs removed in refinement of framework (i.e. received low confidence ratings (<3), low silhouette value (<.25) or were associated with redundant domains and were not allocated elsewhere):'Appraisal', 'Attitudes', 'Certainty of intentions', 'Champions / To champion', 'Change management', 'Commitment', 'Conflict - competing demands', conflicting roles', 'Control of behaviour, material and social environment', 'Coping strategies', 'Crew resource management', 'Direct experience', 'Evaluation', 'Feedback', 'Generating alternatives', 'Hierarchy', 'Illness representations', 'Intention', 'Intrinsic motivation', 'Learning', 'Management commitment', 'Mindsets', 'Moderators of the intention - behaviour gap', 'Negotiation', 'Organisational development', 'Past behaviour', 'Project management', 'Representation of tasks', 'Review', 'Routine / automatic / habit', 'Schemas', 'Sensitisation', 'Supervision', 'Team working', 'Threat'.

(key: *=p<0.05, **=p<0.01, ***=p<0.001, CS / OS Cong. = Closed Sort / Open Sort Congruency (% of original framework constructs included in sort task domains), Non-Italicised Av. S(i) value = excluding constructs in cluster whose values <0.25, Italicised Av. S(i) value = including all constructs forming cluster in that domain, [a] = after applying Hochberg's correction for multiple comparisons within each domain; [b] - Chars. of outcome expectancies = Characteristics of outcome expectancies, Conflict - comp. demands, conf. roles = Conflict - competing demands, conflicting roles, Knowledge = Knowledge (including knowledge of condition /scientific rationale), Mods. of the intention-behaviour gap = Moderators of the intention-behaviour gap, Rewards = Rewards (proximal / distal, valued / not valued, probable / improbable), Trans. model and stages of change = Transtheoretical model and stages of change.
