# Supplementary material for: Validation of the theoretical domains framework for use in behaviour change and implementation research
Source: Implement Sci. 2012 Apr 24;7:37. doi: 10.1186/1748-5908-7-37 (PMC3483008; doi:10.1186/1748-5908-7-37)
Supplement: Additional file 5 — Membership values (%) for each construct to each domain cluster defined in the cluster analysis. [file 1748-5908-7-37-S5.pdf]

Additional file 5. Membership values (%) for each construct to each domain cluster defined in the cluster analysis.

(Values <1% removed; * Construct identified as duplicate in original framework; SPRI = Social / Professional Role and Identity, BaCa = Beliefs about Capabilities, BaCo = Beliefs about Consequences, Rein. = Reinforcement, Int. = Intentions, Opt. = Optimism, MADP = Memory, Attention, and Decision Processes, EC = Environmental Context and Resources, SI = Social Influences, Emot. = Emotion, BR = Behavioural Regulation, Moderators of IB gap = Moderators of Intention-Behaviour Gap)

Original Domain	Construct	Skills	SPRI	BaCa	Opt.	BaCo	Rein.	Int.	Goals	MADP	EC	SI	Emot.	BR
Knowledge	Knowledge	10.31	4.32	5.31	4.29	9.36	2.61	6.14	4.80	18.46	19.21	2.11	1.80	11.27
	Schemas	3.49	1.61	2.84	14.63	21.03	1.23	2.91	1.93	34.58	9.16		1.25	4.35
	Mindset			1.44	59.99	21.32		2.01		3.17	4.03		1.99	1.88
	Illness representations				5.32	85.12				2.60	2.49			
	Procedural knowledge	40.37	2.77	3.82	2.24	3.35	1.71	4.08	2.84	15.93	12.40	1.22	1.42	7.86
Skills	Skills	99.89												
	Competence	99.95												
	Ability	99.35												
	Skill assessment	98.32												
	Practice	13.78	1.44	1.64	1.47	1.34	2.31	6.49	3.45	12.29	11.15			43.21
	Skills development	93.55								1.68	1.10			1.86
	Interpersonal skills	92.61								1.38	1.69			
	Conflict	1.02	4.62	1.50	2.09	2.87	2.74	2.30	1.07	3.80	71.70	2.09		3.38
	Coping strategies	6.55	2.69	2.72	9.16	2.98	2.88	4.38	6.78	14.28	23.13	1.49	4.12	18.82
Social / Professional Role and Identity	Identity			1.80	87.43			1.11		1.44	2.62			1.18
	Professional identity		90.27							1.13	3.07			
	Professional boundaries*		94.28								2.54			
	Professional role*		96.13								1.39			
	Group identity*											99.81		
	Social identity*				1.81					1.73	2.55	87.77		1.26
	Social norms*											99.74		
	Group norms*											99.98		
	Alienation*											99.25		
	Organisational commitment*		95.42								1.30			

Original Domain	Construct	Skills	SPRI	BaCa	Opt.	BaCo	Rein.	Int.	Goals	MADP	EC	SI	Emot.	BR
	Feedback						3.43		1.09	2.03	2.76			86.91
	Change management		89.32							1.18	4.27			1.34
Beliefs about Capabilities	Self efficacy			99.92										
	Control of behaviour	7.59	9.43	11.01	3.63	2.83	1.75	6.98	3.45	10.65	32.41	1.56	1.43	7.30
	Perceived competence			99.92										
	Self confidence			99.63										
	Professional confidence		8.53	78.56	1.10	1.02		1.35		1.53	4.23			1.07
	Empowerment	18.67	6.86	5.41	3.25	2.54	5.00	4.78	5.10	10.76	24.57	2.38	1.19	9.49
	Self-esteem			82.65	10.04					1.11	1.42			
	Perceived behavioural control			99.13										
	Optimism				99.93									
	Pessimism				99.54									
Beliefs about Consequences	Outcome expectancies					99.46								
	Anticipated regret*	1.68	2.06	1.92	11.20	17.83	6.33	6.07	3.51	14.66	21.53	1.49	2.48	9.22
	Appraisal	2.07	1.99	2.14	4.23	9.58	4.16	4.40	2.64	34.29	13.87	1.22	2.30	17.10
	Evaluation	1.80	1.42			1.00	2.00	1.39	1.24	11.59	4.58			72.65
	Review	1.16								3.49	1.74			89.50
	Consequents						87.80	1.41		1.24	3.21			2.81
	Attitudes					99.87								
	Contingencies						91.98				2.94			1.62
	Reinforcement						97.02							1.59
	Punishment						99.63							
	Incentives						99.82							
	Beliefs					99.87								
	Unrealistic optimism				96.72									
	Sensitisation		1.26		1.33		12.18	2.62	1.14	8.22	9.43			59.98
	Salient events / critical incidents	1.42	1.56	1.85	6.81	4.93	2.96	3.25	2.43	7.88	59.15		1.18	5.64

Original Domain	Construct	Skills	SPRI	BaCa	Opt.	BaCo	Rein.	Int.	Goals	MADP	EC	SI	Emot.	BR
	Characteristics of outcome expectancies					94.93								
	Sanctions						96.58				1.66			
	Rewards						99.99							
Motivation and Goals	Intention							95.12	1.54					
	Stability of intentions							95.03			1.06			
	Certainty of intentions							97.85						
	Goals (autonomous / controlled)								97.78					
	Goal / target setting*								99.84					
	Goal priority*								97.82					
	Intrinsic motivation	2.74	2.10	3.84	5.58	4.41	2.02	45.21	4.68	9.10	9.14	1.18	1.76	8.23
	Commitment				1.15			84.82	3.85	1.84	2.86			1.63
	Goals (distal / proximal)								98.99					
	TM&SoC		1.11		1.13	1.30	1.36	78.64	2.40	3.06	4.71			3.20
Memory, Attention and Decision Processes	Memory									94.42	1.22			1.24
	Attention									96.57				
	Attention control									95.31				1.14
	Decision making	2.04	1.44	1.20	2.18	3.82	2.58	4.11	3.29	43.16	10.12			24.31
Environmental Context and Resources	Resources / material resources	3.01	8.56	1.13	1.99	1.33	3.29	2.66	1.38	4.29	66.54	1.14		3.68
	Environmental stressors						2.63	1.35		2.52	86.51			1.59
	Person x environment interaction	1.70	4.65	1.45	3.08	1.51	2.29	3.54	2.18	4.90	61.03	6.67	1.52	5.48
	Knowledge of task environment	9.06	9.63	2.96	2.86	4.17	1.72	4.17	2.01	10.02	41.28	3.55	1.11	7.45
Social Influences	Social support*											99.62		
	Organisational development		99.25											
	Leadership		97.18											
	Team working	1.16	76.10					1.17		2.66	6.42	4.86		3.60
	Group conformity											99.95		
	Organisational culture / climate		99.22											

Original Domain	Construct	Skills	SPRI	BaCa	Opt.	BaCo	Rein.	Int.	Goals	MADP	EC	SI	Emot.	BR
	Social Pressure											99.90		
	Power	5.55	34.08	3.61	2.59	2.04	1.57	3.90	2.05	9.45	16.81	11.23	1.22	5.90
	Hierarchy		69.20					1.04		1.57	5.08	17.58		1.36
	Management commitment		98.00											
	Supervision		92.76								2.84			
	Intergroup Conflict		1.07								1.44	95.87		
	Champions / To champion	2.14	41.33	2.85	2.87	2.11	2.27	7.17	3.19	7.18	17.68	3.53	1.58	6.12
	Social comparisons											98.65		
	Crew resource management		91.79								3.59			
	Negotiation	3.08	23.01	1.98	2.74	2.16	4.13	4.50	5.30	8.01	26.48	5.07	1.38	12.15
	Learning									2.13	1.10			93.52
	Modelling	2.27	4.69	1.33	1.86	2.17	4.90	3.55	2.28	6.99	12.29	16.60	1.19	39.85
Emotion	Affect												98.32	
	Stress												99.02	
	Fear												99.38	
	Burn-out												97.73	
	Cognitive overload / tiredness	1.45	1.41	1.17	2.09	1.72		1.99	1.36	46.90	9.46		27.95	3.05
	Threat	1.72	3.38	2.15	11.47	8.24	7.49	4.26	1.92	9.12	28.64	2.28	13.69	5.64
	Positive / negative affect												99.25	
	Anxiety												99.99	
Behavioural Regulation	Depression												99.96	
	Implementation intention							5.07	87.44	1.27	1.74			2.07
	Action planning							2.34	89.06	1.36	1.87			2.72
	Self-monitoring							1.81	2.18	1.70	2.38			88.67
	Generating alternatives	5.20	1.66	1.39	1.29	1.11	1.73	2.28	6.68	14.53	14.60	1.07		47.73
	Moderators of the IB gap	1.76	2.46	1.55	3.25	2.07	6.44	25.25	19.37	5.10	23.67	1.36	1.07	6.64
	Project management		74.26		1.05			2.20	2.12	3.05	9.72			2.33

Original Domain	Construct	Skills	SPRI	BaCa	Opt.	BaCo	Rein.	Int.	Goals	MADP	EC	SI	Emot.	BR
Nature of the Behaviours	Barriers and facilitators	1.85	1.47	1.96	1.24	1.64	2.64	3.13	3.22	5.14	71.51			4.83
	Routine / automatic / habit	2.44	2.96	1.87	2.81	2.58	10.61	7.16	5.94	21.37	15.81	1.85	1.80	22.80
	Breaking habit	1.87	1.57	1.66	1.49	1.37	3.64	6.17	5.00	8.28	8.35			59.05
	Direct experience	1.04			1.07		2.50	1.54	1.19	5.27	7.78			76.75
	Past behaviour	2.37	2.58	2.01	3.27	5.12	13.37	10.42	4.72	13.24	20.70	1.35	1.46	19.37
	Representation of tasks	3.18	1.79	2.86	4.05	5.18	1.23	3.49	41.71	16.77	10.75			7.08
	Stages of change model		1.04		1.02	1.10	1.11	80.95	2.18	2.67	4.26			2.83
